# NSCLC as the Paradigm of Precision Medicine at Its Finest: The Rise of New Druggable Molecular Targets for Advanced Disease

**DOI:** 10.3390/ijms23126748

**Published:** 2022-06-17

**Authors:** Anna Michelotti, Marco de Scordilli, Elisa Bertoli, Elisa De Carlo, Alessandro Del Conte, Alessandra Bearz

**Affiliations:** 1 Department of Medicine (DAME), University of Udine, 33100 Udine, Italy; anna.michelotti@cro.it (A.M.); marco.descordilli@cro.it (M.d.S.); elisa.bertoli@cro.it (E.B.); 2 Centro di Riferimento Oncologico di Aviano (CRO) IRCCS, Department of Medical Oncology, 33081 Aviano, Italy; elisa.decarlo@cro.it (E.D.C.); alessandro.delconte@cro.it (A.D.C.)

**Keywords:** NSCLC, *MET*, *RET*, *NTRK*, *KRAS*, *HER2*, oncogene-addiction, new targets, precision medicine

## Abstract

Standard treatment for advanced non-small cell lung cancer (NSCLC) historically consisted of systemic cytotoxic chemotherapy until the early 2000s, when precision medicine led to a revolutionary change in the therapeutic scenario. The identification of oncogenic driver mutations in EGFR, ALK and ROS1 rearrangements identified a subset of patients who largely benefit from targeted agents. However, since the proportion of patients with druggable alterations represents a minority, the discovery of new potential driver mutations is still an urgent clinical need. We provide a comprehensive review of the emerging molecular targets in NSCLC and their applications in the advanced setting.

## 1. Introduction: Driver Alterations beyond EGFR, ALK and ROS1: What Do We Know So Far?

Lung cancer represents the leading cause of cancer death, with an estimate of 2.2 million new cases and 1.8 million deaths in 2020 (18% of the total) [1]. The 5-year survival rate from diagnosis is modest, ranging from 10 to 20% globally [2]. Non-small cell lung cancer (NSCLC) accounts for 85% of cancer diagnoses and includes adenocarcinoma (ADC) and squamous cell carcinoma (SCC) as the most frequent histological subtypes [3].

Over the past two decades, following the identification of new druggable targets, the treatment management of NSCLC has considerably changed. The improved understanding of the molecular pathways driving malignancy led to the development of new agents selectively directed to inhibit the signal transduction pathways of protein kinases.

To date, molecular characterization of tumor tissue is essential in defining the treatment strategy of advanced disease and could soon also become a standard of care in early stage, following recent evidence of a survival benefit of targeted agents also in the adjuvant setting [4].

The majority of lung cancer patients presents with advanced disease at diagnosis and oncogene-addiction is found by molecular characterization in less than 25% of cases [5]. Well-established actionable targets in adenocarcinoma include mutations in the gene encoding epidermal growth factor receptor (*EGFR*, 9%) [6,7], anaplastic lymphoma kinase (*ALK*, 3.9%) [8], c-ros oncogene 1 (*ROS1*, 1%) [9] and B-type Raf proto-oncogene (*BRAF^V600E^*, 1%) [10]. In NSCLC harboring an *EGFR* mutation sensitive to tyrosine kinase inhibitors (TKIs), namely exon 19 deletions or L858R point mutation, first line treatment with osimertinib, a third-generation TKI, improved OS by nearly seven months [11]. The preferred option for *ALK*-rearranged tumors is a next generation ALK inhibitor, such as alectinib, brigatinib or lorlatinib. No comparisons have been made between second-generation ALK-inhibitors [12,13,14]. *ROS1* translocation is highly sensitive to both crizotinib and entrectinib, preferring the latter in case of brain metastasis due to a better intracranial penetration [15,16]. For NSCLC carrying a *BRAF^V600E^* mutation, current guidelines recommend a combination of BRAF and MEK inhibitors, namely dabrafenib plus trametinib [17].

Most of the alterations mentioned above are extremely rare in SCC, for which, depending on the levels of PD-L1 expression, immunotherapy or immunochemotherapy represent the main options [5].

Among lung cancer patients, those carrying an oncogenic driver mutation and who received a targeted agent, present an increased median overall survival (OS) compared to patients with non-oncogene addicted disease, providing further evidence for an extensive molecular profiling in order to identify new molecular biomarkers [18]. Moreover, matching a specific target tends to reduce treatment-related adverse events, improving patients’ quality of life and treatment compliance.

Novel potential predictive biomarkers are currently emerging, expanding the landscape of targetable driver mutations.

**Figure 1:**
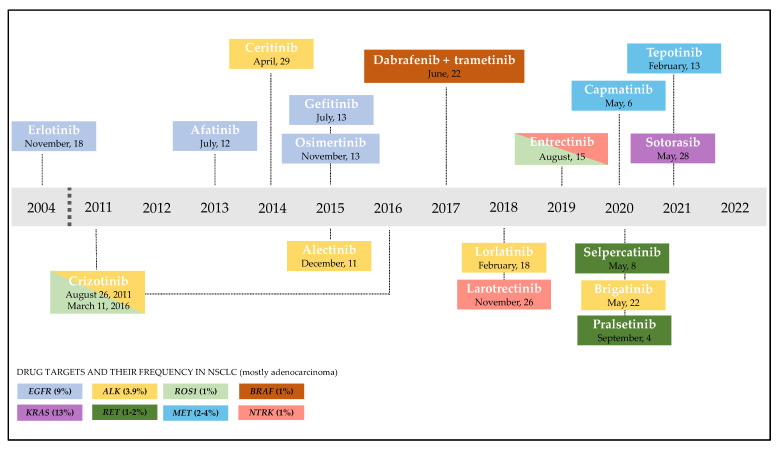
**Timeline of FDA approval of targeted therapies for NSCLC** (the colors are matched between driver alteration and targeted agent). The identification of actionable biomarkers led to significant progress in the treatment of NSCLC. EGFR alterations are detected in approximately 9% of NSCLC patients [6,7] and in the last two decades several agents targeting sensitive mutations received approval from the FDA. The first–generation EGFR–TKI inhibitors, erlotinib and gefitinib, received FDA approval for the treatment of advanced NSCLC in November 2004 and July 2015, respectively. Afatinib is the most studied second–generation inhibitor and received approval in July 2013. Two years later, the third–generation TKI-inhibitor, osimertinib, was initially approved for the treatment of EGFR–T790M mutation positive NSCLC, then in April 2018 it received approval as a first–line treatment for EGFR mutated NSCLC. ALK fusion–positive tumors account for 3.9% of NSCLC adenocarcinomas [8]. Several targeted drugs are available for this subset of patients: the first–generation drug crizotinib was approved in August 2011 and then the FDA expanded its use to treat ROS1–positive patients, a rare subgroup accounting for approximately 1% [9]. Second–generation ALK inhibitors, ceritinib, alectinib and brigatinib, were approved by the FDA between April 2014 and May 2020. The third–generation inhibitor lorlatinib received approval in 2018 for pretreated ALK–positive patients, and later in 2021 for the first–line setting. In June 2017, the FDA approved a combination therapy of dabrafenib and trametinib for BRAFV600E mutation–positive metastatic NSCLC, accounting for 1% of lung cancer patients [10]. NTRK is found in 1% of NSCLC [19,20]. Larotrectinib is a specific NTRK inhibitor approved in 2018 and represents the second tissue–agnostic FDA approval for the treatment of cancer Entrectinib received approval in August 2019 for both treatment of NTRK and ROS1- positive NSCLC. In the last two years, major progress has been made: in 2020 the FDA approved the targeted agents selpercatinib and pralsetinib for RET fusion–positive NSCLC (1–2%) [5,21]; capmatinib and tepotinib received FDA approval for NSCLC harboring a METex14 skipping mutation (2–4%) [22,23] in May 2020 and February 2021, respectively; sotorasib was approved in May 2021 for the treatment of *KRAS^G12C^* mutated NSCLC (approximately 13%) [24] in patients who have received at least one prior systemic therapy.

Currently FDA approved targeted agents are depicted in Figure 1.

Data regarding clinical outcomes of interest achieved with novel agents are summarized in Table 1.

Given the growing body of evidence regarding this topic, the present review mainly focuses on emerging driver alterations for which solid data of clinical relevance deriving from phase 2 or 3 clinical trials are currently available: MET alterations, RET rearrangements, NTRK1/2/3 fusions, *KRAS* mutations and HER2 amplifications or mutations. A literature search using the PubMed Advanced Search tool was conducted including original papers published between January 2010 and April 2022 regarding the above-mentioned alterations of interest. A clinical trials search was conducted using https://www.clinicaltrials.gov/ (accessed on 1 May 2022).

The purpose of this review is to provide a comprehensive overview of the main promising therapeutic targets beyond *EGFR, ALK, ROS1* and *BRAF* and to discuss their potential implications in reshaping the current treatment algorithm for stage advanced NSCLC.

## 2. An Overview of the Main Rising Driver Alterations and Their Clinical Implications

### 2.1. Deregulation of MET Signalling Pathway

Mesenchymal Epithelial Transition (MET) is a proto-oncogene encoding for a tyrosine kinase receptor that binds hepatocyte growth factor (HGF), a protein involved in many crucial processes, including cell survival, migration and invasion [25]. MET alterations are reported in several solid tumors and are considered driver mutations in the carcinogenic process [26]. Three different genomic states can lead to the deregulation of this pathway: gene amplification, mutations and fusions [25]. These alterations result in tyrosine kinase activation and ligand-independent downstream signaling.

MET amplification is found in 1–5% of untreated NSCLC [27,28], and in 5–20% of EGFR mutated tumors with acquired resistance to EGFR-TKIs [29]. Gene amplification can be detected by FISH or NGS panels, however, to date there is no consensus on the actual definition of MET amplification. Studies have tested several cut-offs for FISH positivity: MET– to–chromosome 7 centromere ratio (MET/CEP7) values of 1.8 or higher, 2.0 or higher, 2.2 or higher and 5 or higher [30,31]. Literature data seem to suggest that a FISH MET/CEP7 ratio of 5 or higher could be the optimal cut-off for defining positivity, as a high-level MET amplification strictly correlates with oncogenic-dependance and there is no overlap with other oncogene drivers, and therefore treatment sensitivity [32].

Capmatinib is a highly potent and selective inhibitor of MET receptor that showed in vitro and in vivo activity in preclinical cancer models with diverse types of MET aberrations [33]. Its clinical efficacy and safety were analyzed in the prospective, international, multicohort, open-label, phase 2 trial, GEOMETRY mono-1. The trial included naïve or pretreated patients with stage IIIB and IV NSCLC with no EGFR mutation or ALK fusion, tested positive for MET amplification or MET exon 14 skipping mutation. MET amplified NSCLCs were classified according to gene copy number (GCN) in tumor tissue as follows: GCN less than 4, 4–5, 6–9 and 10 or higher. Notably, limited efficacy was registered in patients with a GCN less than 10, with an overall response rate (ORR) ranging from 7–12% and a median progression-free survival (PFS) from 2.7–3.6 months. Among tumors with a GCN of 10 or higher, an objective response was registered in 29% of pretreated, and in 40% of treatment naïve patients, although the overall response was lower than the prespecified threshold set for a clinically relevant activity [34].

Treatment options for advanced NSCLC with MET amplification also include the MET inhibitor, crizotinib. Efficacy data is derived from small cohorts of phase 1 and 2 trials. The phase 1 PROFILE 1001 trial enrolled 38 MET positive (with a MET/CEP7 ratio ≥ 1.8) NSCLC patients. Consistent with the findings of the GEOMETRY mono-1 trial, response rates were greater in patients with high MET amplification: ORR of 38% in MET/CEP7 ratio ≥4.0, 14.3% in MET/CEP7 ratio 2–4 and 33.3% in MET/CEP7 ratio >1.8, with a median PFS of 6.7, 1.9 and 1.8 months, respectively [35]. Similarly, the phase 2 METROS trial enrolled a cohort of 26 MET deregulated NSCLC patients, 16 of them with a MET amplification, 9 with a MET exon 14 (METex14) skipping mutation and 1 patient with cooccurrence of the two alterations. The ORR was 27% (all partial responses), stable disease was registered in 42% of cases, with a global disease control rate (DCR) of 69%. Although treatment response rates were promising for a pretreated population, survival outcomes were poor: at a follow up of 21 months, median PFS and OS were 4.4 and 5.4 months, respectively [36].

To date, neither capmatinib nor crizotinib have been approved by the FDA or EMA for pretreated NSCLC with high-level MET amplification.

Exon 14 encodes the 47-amino acid juxtamembrane domain of the MET receptor, a key regulatory region preventing MET overexpression and thus oversignalling [37]. The genomic events underlying the mis-splicing of MET exon 14 are complex and include several types of alterations, such as point mutations, insertions or deletions. The specific mechanism of carcinogenesis has not been fully elucidated yet, however, the loss of this region results in an impaired MET receptor degradation and aberrant activation of the signaling pathway [38].

In NSCLC, METex14 is observed in approximately 2–4% of cases [22,39] as tested by DNA or RNA NGS. Given the diversity of alterations that may lead to MET exon 14 skipping and the potential location of these alterations in the MET gene, the optimal testing technique is still matter of debate [23]. The phase 2 trial GEOMETRY mono-1, included 97 patients tested positive for a MET exon 14 skipping mutation. In this subset of patients, capmatinib showed substantial antitumor activity. An overall response was observed in 41% of patients who had received one or two prior lines of therapy and in 68% of treatment-naïve patients; the median duration of response (DoR) was 9.7 months and 12.6 months, respectively. The median PFS was 5.4 months in pretreated and 12.4 months in untreated patients [34]. Notably, responses to capmatinib were rapid, with the majority of patients showing response at the first radiological evaluation. No difference in response to the study drug was observed according to the specific genetic alteration causing METex14 skipping mutation.

Tepotinib activity was assessed in the multi-cohort, phase 2 VISION trial, conducted on advanced NSCLC patients with evidence of METex14 skipping mutation detected on tissue or liquid biopsy, who received up to two courses of previous therapy for metastatic disease. Globally, the response rate confirmed by independent central review (ICR) (primary study endpoint) was 46%, with a median duration of response of 11 months. The investigator-assessed response rate was 56%, similar to previous lines of therapy [40].

Following the results of the GEOMETRY mono-1 and VISION trials, the FDA approved capmatinib and tepotinib for adult patients with advanced NSCLC harboring a METex14 skipping mutation on 6 May 2020, and 13 February 2021, respectively [41]. On 16 February 2022, tepotinib received marketing authorization valid throughout the European Union [42]. On 22 April 2022, the Committee for Medicinal Products for Human Use (CHMP) adopted a positive opinion, recommending the granting of capmatinib authorization, however European Medical Agency (EMA) final approval is still awaited [43].

The safety profile of capmatinib and tepotinib was similar, and the most common adverse events (AEs) recurring in ≥10% of patients reported in clinical trials included peripheral edema, nausea, vomiting and increased blood creatinine level [34,40].

A better understanding of MET deregulation mechanisms contributed in recent years to the development of additional strategies: highly selective oral/intravenous MET inhibitors, combination therapies, humanized antibodies and antibody drug conjugates (ADC). Savolitinib is an oral selective MET inhibitor that showed promising results in a phase 2 trial on METex14 mutated lung sarcomatoid carcinomas with an ORR of 49% by ICR [44] and is currently under evaluation in combination with osimertinib in patients affected by MET-altered NSCLC with acquired resistance to EGFR-TKIs (NCT05015608, NCT05163249). Other molecules for MET-altered NSCLC are under investigation in early phase clinical trials (Table 2) including: glumetinib and APL-101 (oral MET inhibitors); SAR125844, a MET inhibitor administered intravenously; glesatinib (MGCD265) with activity against MET; VEGFR1/2/3; RON; TIE-2; and elzovantinib (TPX-022), a potent MET/CSF1R/SRC inhibitor. Current research is also directed to test the efficacy of biopharmaceutical drugs, such as Sym015, a mixture of two humanized IgG1 antibodies, directed against nonoverlapping epitopes of the MET ectodomain [45] and ADC telisotuzumab vedotin [46].

### 2.2. RET Rearrangements

Rearranged during transfection (RET) gene encodes for a transmembrane receptor of the tyrosine protein kinase family that binds the glial cell-line derived neurotrophic factor [47]. In human cancer, oncogenic activation is mainly a consequence of cytogenetic rearrangements, and the derived chimeric genes determine the transcription of an aberrant receptor with dysregulation of downstream processes, such as cell proliferation, migration and survival [47]. RET fusion positive NSCLCs are an extremely rare subgroup of patients (1–2%) first described in 2012 [5,21]. Clinicians must be aware of the importance of the adoption of adequate molecular testing techniques and the use of NGS should be preferred over FISH or real-time PCR due to its higher sensitivity [48].

First attempts to target RET fusions included the use of several oral multikinase inhibitors with proven clinical efficacy in other solid tumors (e.g., cabozantinib, vandetanib and lenvatinib). The retrospective international registry GLORY represents the largest database of RET-rearranged lung cancers and data were collected with the aim to document outcomes of patients treated with these molecules. Globally, response rates reported ranged from 18–37%, with a median PFS of 2.3 months and a median OS of 6.8 months [49]. Outcomes are disappointing compared with the activity of targeted therapy in other genomic subsets of lung cancer, and, as consequence of the off-target side-effects, treatment is burdened by excessive toxicities.

The development of RET selective inhibitors represented an effective strategy to potentially overcome the poor results obtained with multikinase inhibitors while also reducing treatment-related adverse events (AEs). In this setting, selpercatinib is a novel agent that selectively binds to and targets various RET mutants and RET-containing fusion products. The phase 1/2 trial LIBRETTO-001, designed to test the safety and activity of selpercatinib, enrolled 105 patients with RET fusion-positive advanced NSCLC who had previously received at least a platinum-based chemotherapy. In 36% of cases, brain metastasis were present at baseline. Moreover, the study population was heavily pretreated with a median of three previous systemic lines of therapy and almost half of patients already exposed to a multitargeted kinase inhibitors with anti-RET activity. The study met its primary endpoint registering a 64% of ORR confirmed by ICR, mostly partial responses [50]. Among patients with measurable brain involvement, the objective intracranial response by ICR was 91%. Selpercatinib was effective regardless of previous therapy or specific RET fusion partner.

The ARROW trial is a multi-cohort, international, open-label, phase 1/2 study designed to define the maximum tolerated and recommended dose of the oral selective RET inhibitor, pralsetinib, and to test its clinical activity and safety. Overall, 92 patients treated with a median of two previous lines and 29 treatment naïve patients who were not candidates for standard platinum-therapies, were included in the phase 2 study. Notably, in 41% of cases, baseline central nervous system (CNS) involvement was documented. Response rates were remarkable in both the pretreated and the treatment naïve group, with an ORR of 61% and 70%, respectively. Shrinkage of intracranial metastases was seen in all patients with measurable intracranial metastases at baseline. At a median follow up of 14.7 months, in previously treated patients median PFS was 17.1 months and median OS not reached. At a median follow-up of 11.6 months, in untreated patients median PFS and median OS were 9.1 and not reached, respectively [51]. As seen for selpercatinib, pralsetinib activity was not affected by previous treatments received, including anti-PD1, anti-PDL1 or multikinase inhibitors, or by RET diverse fusion partners.

Overall, 93% of patients had treatment-related AEs, most common G ≥ 3 AEs were neutropenia (18%), anemia (10%), hypertension (11%) and pneumonia (10%). Most common G1-2 AEs (reported in ≥10% of patients) included hematological toxicity (neutropenia, anemia, leucopenia), AST/ALT increase, asthenia, constipation, hypertension, dysgeusia and increased blood creatinine [51]. Despite the limitation of cross-trial comparisons, the overall frequency of adverse events with pralsetinib was comparable to selpercatinib [50].

Following the results of LIBRETTO-001 and ARROW trials, the FDA granted approval of pralsetinib and selpercatinib for the treatment of RET fusion-positive NSCLC [52,53]. Selpercatinib and pralsetinib received a conditional marketing authorization from the EMA in February and November 2021, respectively [54,55]. Therefore, in the European Union these two targeted agents are still under additional monitoring.

Currently, the next generation of RET-TKIs is under exploration in early phase clinical trials (Table 3). Of particular interest is TPX-0046, a third generation orally bioavailable RET/SRC kinase inhibitor, with preliminary evidence of activity against a range of RET fusions and resistance mutations in tumors models [56]. BOS172738 is an investigational, potent, next generation selective oral RET kinase inhibitor with reported clinical activity and a good safety profile in a phase 1 trial [57]. As resistance is a major challenge for RET fusion-positive NSCLC [58], the development of next generation RET inhibitors with activity against acquired mutations could represent an effective treatment option after progression.

### 2.3. NTRK1, NTRK2, NTRK3 Fusions

The Neurotrophic Tropomyosin Receptor Kinase (NTRK1, NTRK2, NTRK3) gene family encodes Tropomyosin Receptor Kinases (TRKA, TRKB, TRKC, respectively) [59,60,61,62].

Physiologically expressed in neuronal cells, these three transmembrane proteins, binding neurotrophic factors, are fundamental to the development and function of the nervous system. Ligand binding causes the oligomerization of these receptors’ kinases, leading to final activation of intracytoplasmic pathways (MAPK, PI3K and PLC-γ) involved in cell proliferation, differentiation and survival [61,62]. Alterations in TRK pathways are involved both in nervous system diseases (depression, epilepsy, or neuropathic pain, etc.) [63] and cancers [59,60,61,62]. The main oncogenic gene alteration in cancer is NTRK gene fusion, producing overexpressed or constitutively activated fusion receptor kinases [64,65]. Alternative oncogenic mechanisms include TrkA alternative splicing, implicated in neuroblastoma, and in-frame deletion of NTRK1, related to acute myeloid leukemia [61]. NTRK fusions represent a rare therapeutic target in solid neoplasms, in NSCLC their frequency is reported from 0.1% up to 1% [19,20] and generally are mutually exclusive with other oncogene alterations. NTRK fusions have also been described as a mechanism of acquired resistance to EGFR TKIs in patients with EGFR mutated NSCLC [66].

*NTRK*1/2/3 fusion gene detection is independent of tumor type and follows the European Society for Medical Oncology (ESMO) recommendations [67]. Molecular testing includes two alternative methods: screening with immunohistochemistry (IHC), followed by next generation sequency (NGS), if possible, RNA-based NGS; or NGS techniques confirmed by IHC in positive cases [67].

Several targeted drugs for NRTK rearrangements are under current development and some of these have been introduced in clinical practice thanks to different basket trials. Among TRK inhibitors, multi-kinase inhibitors also present anti-TRK activity (entrectinib, repotrectinib, selirectinib, taletrectinib, etc.), while larotrectinib is a member of selective inhibitors [62,67].

Larotrectinib is characterized by high selectivity for TRKA, TRKB and TRKC and it is the first oral pan-TRK inhibitor to receive tissue-agnostic FDA approval (November 2018) for advanced NTRK fusion-positive solid tumors, based on the results on 55 patients of three multicenter, open-label, single-arm clinical trials (LOXO-TRK-14001, SCOUT and NAVIGATE trials) [68]. Larotrectinib also received a conditional marketing authorization by the EMA in September 2019 with the same therapeutic indications [69].

In April 2020, Hong and colleagues [70] presented updated results of these three trials: among 159 patients, 12 patients had NSCLC with an ORR of 75%, consistent with ORR in the overall population (79%). Considering survival in all patients, a median OS of 44.4 months and a median PFS of 28.3 months were reported. In the safety analysis, larotrectinib was well tolerated with grade 4 adverse events in 1% and grade 3 in 13% of patients (grade 3–4: neutropenia in 2%, anemia in 2% and elevation of aspartate aminotransferase, AST, or alanine aminotransferase, ALT, in 3%), without treatment-related deaths. Among AEs of all grades, the most frequent ones were fatigue in 30% of patients, increase of liver aminotransferase in 28% and cough in 27%.

Entrectinib, as a member of the oral multi-kinase inhibitors, also inhibits (in addition to TRKA, TRKB, TRKC) ROS1 and ALK. Its peculiarity is its high ability to cross the blood–brain barrier, showing activity in patients with CNS disease [62,67]. In August 2019, Entrectinib reached FDA accelerated approval for advanced solid tumors with NTKR fusions and for metastatic ROS1-positive NSCLC based on the results of three multicenter, single-arm, clinical trials (STARTRK-1, STARTRK-2, ALKA-372-001) [71]. In July 2020, entrectinib received a conditional marketing authorization for both NTRK gene fusion solid tumors and ROS1-positive advanced NSCLC, addressing a major unmet medical need in this subset of patients [72].

Recently, an updated integrated analysis of three studies focused on ROS1 fusion-positive NSCLC showed an ORR of 67%, a median DoR of 15.7 months, a median PFS of 15.7 months and a median OS was not estimable [73]. Considering patients with CNS metastases, the intracranial ORR was 79% (95% CI, 58–93%), intracranial DoR was 12.9 months and median intracranial PFS was 12.0 months. The updated safety analysis was consistent with the primary one: most adverse events were grade 1–2 (dysgeusia 43%, dizziness 34%, constipation 31%), while grade 3 adverse events were weight increase (8%), ALT elevation (3%) and diarrhea (3%). Grade 4 adverse events were reported in 3% of patients (hyperuricemia, limbic encephalitis, anorectal disorder, hypertriglyceridemia, myocarditis, blood creatine phosphokinase myocardial band increase, anorectal disorder). Both for larotrectinib and entrectinib, dose modifications were able to control treatment-related AEs.

Another oral multi-kinase inhibitor is taletrectinib (DS-6051b/AB-106), which presents a high selectivity for ROS1/NTRK fusion genes. The efficacy of this TRK inhibitor in ROS1+ NSCLC emerged in two phase 1 studies (U101, conducted in United States, and J102, in Japan) [74]. With a median follow up of 14.9 months, in ROS1 TKI-naive patients an ORR of 67% was detected, while in crizotinib pretreated patients ORR was 33%. Considering survival, in the first group PFS was 29.1 months, and it was 14.2 months in the second group. Taletrectinib presented a manageable safety profile: most reported AEs were ALT and AST increase (both 73%), and nausea and diarrhea (both 50%), of which grade ≥3 were ALT and AST increase (18% and 9%, respectively) and diarrhea (5%). A multicenter, phase 2 clinical trial (TRUST, NCT04395677) is currently ongoing to evaluate the efficacy of taletrectinib in Chinese ROS1-positive NSCLC patients, while the TRUST-II trial is the ongoing global study (NCT04919811). At ASCO 2021, Zhou et al. [75] showed that all enrolled Chinese patients at the data cutoff presented a response to the treatment with an ORR of 100% (95% CI, 72–100%) and a safety profile consistent with phase 1 data.

Acquired resistance is still an inevitable circumstance in patients treated with TRK inhibitors, despite the durable and terrific duration of response, regardless of tumor type [67,76]. This acquired resistance is often due to the appearance of new NTRK mutations [67]. Repotrectinib (TPX-0005) and selirectinib (LOXO-195) are next generation TRK inhibitors designed to overcome resistance to first-generation TRK inhibitors. Repotrectinib is highly selective and active for ALK, ROS1 and NTRK, thus potentially overcoming acquired resistance [77]. The TRIDENT-1 trial is the ongoing phase 1/2 study of repotrectinib for ALK/ROS1/NTRK fusion gene-positive NSCLC (NCT03093116). Selirectinib’s chemical structure is similar to larotrectinib, apart from the more compact form. In a preclinical study, selirectinib showed resistance to secondary resistance mutations in the TRK kinase domain [76]. In 31 patients who received selirectinib after progression to a TRK inhibitor (mainly larotrectinib), the ORR was 34%, while it was 45% in patients with secondary resistance mutations [78]. An ongoing phase 1/2 study to test efficacy and safety of selirectinib is active, not recruiting (NCT03215511).

### 2.4. KRAS Mutations

The family of rat sarcoma oncogenes (RAS) includes the isoforms Kirsten rat sarcoma (*KRAS*), neuroblastoma rat sarcoma (*NRAS*) and the Harvey rat sarcoma (*HRAS*). Ras proteins activate signaling pathways controlling cell proliferation, differentiation and survival [79]. *KRAS* accounts for 85% of RAS mutations observed in human cancer and, given that RAS is the most frequently mutated oncogene, it is the most prevalent genomic driver event in NSCLC, present in up to 35% of lung cancers [80,81]. Notably, *KRAS* mutations are more common in the adenocarcinoma histotype than in squamous NSCLC (20–40% and 5%, respectively) and most frequently found in smokers (30%) vs. non-smokers (11%) and in the Caucasian vs. Asian population (26% and 11%, respectively) [82,83]. The *KRAS* p.G12C single-nucleotide variant, with glycine replaced by cysteine at codon 12, is the most recurrent variant in NSCLC, with a prevalence of nearly 13% in adenocarcinoma histotype [24]. It represents 39% of *KRAS* mutations, followed by G12V (21%) and G12D (17%) [84].

Sotorasib is a small molecule that specifically and irreversibly inhibits *KRAS^G12C^* from binding covalently to a pocket present only in the inactive GDP-bound conformation, trapping *KRAS^G12C^* in the inactive state and hindering *KRAS* oncogenic signaling [85]. In the phase 1/2 CodeBreak 100 trial, sotorasib monotherapy was evaluated in patients with locally advanced or metastatic *KRAS*^G12C^ mutated NSCLC. At a median follow-up of 15.3 months, ORR (primary endpoint) was 37.1% and DCR 80.6%, with a median time to response of 1.4 months (range, 1.2–10.1), a median duration of response of 11.1 months and a PFS of 6.8 months. The most common adverse events were diarrhea, nausea, fatigue, arthralgia and increase in the transaminases [86]. In May 2021, the FDA approved sotorasib as the first targeted agent for *KRAS^G12C^* mutated NSCLC, pretreated with at least one prior systemic therapy. Sotorasib has also been given conditional authorization by the EMA in January 2022 in the same therapeutic setting [87]. This is the first authorized targeted therapy for tumors with *KRAS* mutation. Currently, the phase 3 trial, CodeBreak 200 is comparing sotorasib with docetaxel in patients with *KRAS^G12C^* mutated NSCLC in progression to a platinum-based doublet chemotherapy and a checkpoint inhibitor (NCT04303780).

Adagrasib is another highly selective, small-molecule, covalent inhibitor of *KRAS^G12C^*, with a longer half-life than sotorasib [88]. In the phase 1 trial, KRYSTAL-1, adagrasib was well tolerated and exhibited antitumor activity with 53.3% of partial responses, a median DoR of 16.4 months and a median PFS of 11.1 months [89]. Recently published data on the registrational phase 2 cohort of the KRYSTAL-1 trial, shows that heavily pretreated patients achieved an ORR of 42.9% with adagrasib, with a median DoR of 8.5 months, median PFS and OS of 6.5 months and 12.6 months, respectively [90]. Similarly to sotorasib, most common treatment-related adverse events (of any grade) were nausea, diarrhea, vomiting and fatigue [89]. Based on the findings from the phase 2 KRYSTAL-1 trial, adagrasib received breakthrough therapy designation from the FDA for patients with advanced NSCLC harboring the *KRAS^G12C^* mutation, and a new drug application (NDA) was filed in February 2022. A marketing authorization application has also been submitted to the EMA seeking adagrasib approval in May 2022 [91]. The ongoing confirmatory phase 3 KRYSTAL-12 trial is evaluating the use of adagrasib compared with docetaxel in patients with *KRAS^G12C^*-mutated NSCLC in a second line setting (NCT04685135).

Various *KRAS* inhibitors are currently under investigation (e.g., GDC-6036/RG6330; NCT04449874; JDQ443; NCT04699188; D-1553; NCT04585035; JAB-21822; NCT05276726; RMC-6236, NCT05379985; LY3537982; NCT04956640), as well as combination therapy. (Table 4). Other strategies under evaluation for *KRAS* mutant NSCLC include the inhibition of downstream signaling pathways with MEK inhibitors (NCT04967079, NCT03170206), either as monotherapy or combined with other molecules (NCT03170206, NCT04735068).

### 2.5. HER2 Mutations and Amplifications

Human epidermal growth factor receptor 2 (HER2, also known as ERBB2) is an EGFR family receptor tyrosine kinase that binds tightly with other EGFR family members forming a heterodimer and activating several downstream signaling pathways supporting cell proliferation and survival [92]. Since its discovery, *HER2* has promptly emerged as a relevant oncogenic target in several solid tumors [93,94,95]. *HER2* deregulation can occur through various mechanisms, mainly gene amplification that leads to receptor overexpression, as well as activating kinase domain mutations. No clear association has been reported between *HER2* mutations and amplification.

Different therapeutic strategies are available for HER2-directed therapy: monoclonal antibody trastuzumab in combination with cytotoxic regimens; antibody drug conjugates (ADCs), such as ado-trastuzumab emtansine (T-DM1) and fam-trastuzumab deruxtecan (T-DXd, DS-8201a); panHER inhibitors (afatinib, neratinib, dacomitinib); and novel TKIs (poziotinib, pyrotinib and mobocertinib).

Following the success of anti-HER2 directed therapy in breast and gastroesophageal cancers, a growing interest emerged in a potential clinical application in NSCLC. However, *HER2* amplification and overexpression has been studied as a predictive biomarker of response to targeted therapy in several trials with modest results.

Trastuzumab, in combination with taxanes or platinum-based chemotherapy, showed limited activity in phase 2 trials conducted on HER2 positive pretreated advanced NSCLC. Response rates were similar to those expected with chemotherapy alone, ranging from 8% to 25% across studies with a lack of substantial improvement in survival outcomes [96,97,98]. Likewise, phase 2 trials testing the ADC T-DM1 failed their primary endpoint, with ORRs <6% in the global study populations, signals of minimal activity were restricted to the HER2 amplified IHC 3+ subgroup [99,100].

Limited clinical benefit observed in HER2 amplified NSCLC patients led to the conclusion that overexpression and amplification seem to be a suboptimal biomarker for patients’ stratification, shifting the attention to *HER2* mutations as valuable targets.

In NSCLC, the presence of *HER2* mutations, specifically exon 20 in-frame insertions, identifies a small subset of patients accounting for 1% to 3% of cases [5]. *HER2* gene mutations were first described in a cohort of 120 lung cancer patients in 2004 and seem to be mutually exclusive of other driver mutations, thus confirming their oncogene-addiction potential [101]. The hypothesis of *HER2* mutations being a more reliable biomarker to drive therapy seems to find confirmation in the clinical outcomes of patients treated with ADCs. In a phased 2 basket trial, including a heavily pretreated cohort of *HER2* mutant advanced NSCLC, T-DM1 produced an ORR of 44% with a median PFS of 5 months. 39% of patients had stable disease and durable disease control for up to 11 months. Regarding molecular characterization, responders were seen across all *HER2* mutation subtypes, including exon 20 insertions and transmembrane and extracellular domain point mutations. The concurrent *HER2* amplification observed in 11% of patients did not affect response to treatment [102]. Targeting *HER2* mutations with the next generation ADC trastuzumab, deruxtecan, resulted in even higher response rates, with a confirmed ORR of 55%, a median PFS of 8.2 months and a median OS of 17.8 months [103]. Consistent with previous data, in the DESTINY-Lung01 trial, responses were documented across all types of *HER2* mutations included in the study. A randomized, open-label, phase 3 trial (DESTINY-Lung04, NCT05048797) is currently evaluating T-Dxd compared to standard of care in NSCLC patients harboring *HER2* exon 19 or 20 mutations in a first line setting.

Of note, at the interim analysis of the HER2 overexpressing cohort of the DESTINY-Lung01 trial, T-DXd demonstrated preliminary evidence of activity with 25% of response rate and a median PFS of 5.4 months [104]. Final analyses are awaited.

Case reports, retrospective and early-phase prospective clinical trials registered modest response rates in pretreated *HER2* mutant tumors receiving afatinib, dacomitinib, or neratinib monotherapy. Reported ORRs varied from 0–19% [105,106,107,108,109,110].

While pan-HER2 inhibitors did not confirm the expected potential for disease control, novel and more selective HER2 TKIs showed a promising activity in *HER2* exon20 insertion mutant pre-treated NSCLC patients. Poziotinib, pyrotinib and mobocertinib could become valid options after progression to standard treatments, pending further confirmatory data [111,112,113,114,115,116]. Two ongoing, randomized, phase 3 trials are testing the safety and efficacy of pyrotinib and poziotinib compared to docetaxel in HER2 mutated advanced NSCLC in progression to first line platinum-based chemotherapy (NCT04447118, NCT05378763).

Molecules under investigation in ongoing phase 1 clinical trials include: HER2 exon 20 inhibitors BAY2927088 (NCT05099172) and BI 1,810,631 (NCT04886804); ADCs, such as SBT6050 (NCT05091528, NCT04460456) and A166 (NCT03602079); BDC-1001 a HER2-targeting immune-stimulating antibody conjugate (ISAC) (NCT04278144); and ZW25, a HER2-targeted bispecific humanized antibody (NCT02892123).

To date, none of the above mentioned anti-HER2 agents have received approval from regulatory agencies (nor the FDA or EMA). Patients with a documented *HER2* mutation should be enrolled in clinical trials, especially in case of good performance status and in absence of other available targeted therapy options.

### 2.6. Current Evidence and Ongoing Trials Testing Combination Strategies

The potential benefit of combining new pharmacological agents in oncogene-driven NSCLC patients is still a subject of study. Several clinical trials have started to associate both novel targeted therapies and immune checkpoint inhibitors (ICIs). Little data is available yet, and clinical studies have mainly focused on EGFR and ALK-TKIs.

Regarding possible associations, including drugs specific to new driver alterations, a combination therapy may also represent a mechanism to overcome pharmacological resistance. For example, *MET* amplification is reported in 5–26% of EGFR mutated NSCLC with acquired resistance to EGFR-TKI treatment, representing a potential actionable target after progression to first line treatment [117,118]. Dual targeting EGFR-MET with osimertinib in combination with savolitinib in MET-amplified, EGFR mutation-positive NSCLC progressed to first line EGFR-TKI showed promising antitumor activity at the interim analysis of the phase 1b SAVANNAH trial [119]. In the same setting, the efficacy of the association of tepotinib and gefitinib was tested in the phase 1b/2 INSIGHT trial, registering an improved PFS and OS in patients with high *MET* overexpression or amplification [120]. Moreover, cohort A of a phase 1/2 trial enrolled a small group of 12 patients with EGFR mutated, *MET*-amplified or with *MET*ex14 skipping mutation tumors, treated with capmatinib plus erlotinib; four out of nine patients displayed a durable benefit with the combination therapy [121]. Another phase Ib/II trial assessed promising results with the combination of capmatinib and gefitinib, with an ORR of 47% in patients with *MET* amplification [122]. Capmatinib is being tested in association with osimertinib in a phase III trial (NCT04816214). The combination of capmatinib with ICIs is also being currently investigated in several clinical trials. In a phase II trial, capmatinib plus pembrolizumab did not show a benefit in comparison to pembrolizumab alone as first line treatment in MET-unselected NSCLC patients with PD-L1 ≥ 50% [123]. The combination of capmatinib with ICIs is also being studied after first line failure (NCT03647488).

Of interest, *KRAS* inhibition combined with SHP2 (Src homology phosphatase 2), on which mutated *KRAS^G12C^* cells are dependent [88], is a strategy currently in study (NCT04330664, NCT04185883), as well as dual inhibition of *EGFR* and *KRAS*. Both KRYSTAL-1 and CodeBreak 101 trials included an arm combining respectively adagrasib and sotorasib with afatinib (EGFR TKI) or with a pan-ErbB inhibitor (NCT01859026, NCT04185883). Moreover, preclinical data suggest a synergistic effect of *KRAS* with ICIs [85] [124]. Several trials are evaluating these combinations (e.g., NCT03785249, NCT04185883, NCT04613596).

Other clinical trials are currently exploring the association of new targeted therapies either with other targeted agents (NCT02258607) or chemotherapy (NCT05364645, NCT02642042).

## 3. Discussion

Over the last two decades, substantial advances have been made in the discovery of critical genetic alterations driving the carcinogenic process in NSCLC.

Given the costs in terms of toxicity and quality of life impairment for patients, to determine if there is an actual benefit from therapeutic advances represents a crucial point. Major evidence supporting a survival benefit of targeted therapies on lung cancer patients derives from a large study conducted in the United States (US) [18]. In the overall population, a decrease in lung cancer-related mortality during the study timeframe (2001–2016) has been registered. Looking closely at histological subtypes, for NSCLC, mortality declined from 2006 to 2013, when it underwent an acceleration, shortly after the introduction of routine molecular testing for EGFR and ALK and targeted therapies became available. Moreover, mortality decreased faster than it did for the NSCLC subtype, while for SCLC appeared to be steady from 2006–2016 and shows a similar decline in incidence [18]. These observations seem to suggest that a reduction in incidence coupled with treatment advances, in particular the rise of targeted therapies, is likely responsible for the reduced mortality in NSCLC subgroup. Despite strong evidence, this topic is still a matter of debate [125,126] and further confirmatory studies evaluating the effect of novel targeted therapies, subtype-specific mortality trends and screening, are needed.

It is known that oncogene-addicted disease is more frequent in, but not exclusive to, non-smokers, except for the mutation of *KRAS*. An area of interest is whether the target therapy has the same efficacy in smokers as non-smokers. Concerning FDA approved new targeted molecules, data are scarce. In the Geometry-mono1 trial, 3–4% and 32–39% of patients with MET Exon 14 Skipping Mutation patients and 53–78% and 10–30% with MET amplification were former active smokers or smokers, respectively [34]. In the post-hoc subgroup analysis of ORR, in METex14 patients, the ORR was 45% vs. 34.5% in the pretreated cohort and 72.2% vs. 60% in the naïve one in never smoker and smoker, respectively; in the MET amplification GCN10 pretreated cohort, ORR was 0% vs. 31% and in the naïve cohort 50% vs. 38.5% in never smoker and smoker patients [34]. A consistency of treatment effect achieved with capmatinib, despite a small sample size, was seen in all prespecified subgroups, including smoking status [34]. In the efficacy population of the VISION trial, 46% of patients were current or former smokers [40]. In the LIBRETTO-001 study testing selpercatinib and ARROW trial of pralsetinib, the percentage of patients with smoking history was 35% and 45% [50], 29% and 26% [51] in pretreated and treatment naïve cohorts, respectively. Sotorasib efficacy was tested in a phase 2 trial including 92.2% active or former smokers [86]. However, in none of the above-mentioned trials, except Geometry-mono1, was a subgroup analysis according to smoking status planned. In the pooled analysis of trials testing larotrectinib and entrectinib, data regarding smoking history of patients were not available [16,70]. This area is therefore of interest for future research.

To date, the standard approach for patients with advanced disease is based on tumor molecular characterization to select patients with an oncogene-addicted disease. The role of molecular pathology has therefore evolved from a merely diagnostic purpose to an essential tool to drive therapeutic decisions though the integration of morphologic and molecular information. Due to the increasing complexity of the therapeutic scenario and drug approval criteria, defining the proper method and platform for biomarker testing should be a priority to limit the probability of missing the detection of a driver alteration, thus potentially depriving patients of a highly effective treatment [127]. While testing for gene mutations or rearrangements has a binary outcome (detected/not detected), for gene copy number alterations the definition of the appropriate technique and threshold for positivity is challenging and several cut-offs, as for *MET* amplification, have been proposed. The success of drug testing in clinical trials strictly depends on the identification of an adequate molecular biomarker for patients’ selection. This can explain more than a decade of negative clinical trials testing anti-HER2 targeted agents in *HER2* positive NSCLC. As for breast and gastroesophageal cancers, first studies were conducted on advanced lung cancers with *HER2* amplification or overexpression determined by immunohistochemistry (IHC) with discouraging results. Further research then identified *HER2* activating mutations as promising biomarkers to drive patients’ eligibility with improved outcomes in clinical trials [103].

While molecular characterization represents the current standard of care for NSCLC adenocarcinoma, squamous cells lung cancer genetic profiling, according to current guidelines, should be limited to patients with clinical features associated with a higher prevalence of mutations, such as a lack of smoking history [128]. The reported frequency of *EGFR* mutation in SCC varies from 4.5–9%, however these high rates could be due to the presence of a mixed adenosquamous histology [129,130]. Genomic data analyses evidenced that *EGFR* and *KRAS* mutations, the most common aberrations in lung adenocarcinoma, are extremely rare in pure SCC, while alterations in the FGFR kinase family, PIK3CA, CDKN2A and RB1 pathways are common [131,132]. *ALK*, *ROS1* and *RET* gene rearrangements were not detected in a cohort of more than 200 SCC patients [133], similarly, the estimated prevalence of *BRAF* mutation is <1% [134]. *MET* amplification or exon 14 skipping have a reported frequency of approximately 5% in SCC [135]. As for other well-established driver alterations, *HER2* mutation and amplification are less common in SCC compared to lung adenocarcinoma [136], while the prevalence of *NTRK1/2/3* gene fusions do not vary across histologies [19].

Oncogenic driver alterations tend to present a peculiar molecular epidemiology and gender prevalence. The presence of *MET* exon14 skipping mutation and *MET* amplification can be typically found in elderly female patients with non-smoking history and are more frequent in adenocarcinoma or sarcomatoid histology [39,137,138,139]. Similarly, *HER2* aberrations, particularly *HER2* mutations, are more frequent in never smoker women, affected by NSCLC of adenocarcinoma histology [140,141,142,143]. Taken together, *KRAS* mutations recur more often in smokers versus nonsmokers and in western patients [83]. However, the clinically relevant mutation, *KRAS^G12C,^* displays a higher frequency in women of younger age and lesser smoking history [84]. *RET* fusion gene occurs predominantly in young (<60 years), non-smoker patients and gender prevalence seems to have a geographic correlation: more frequent in European males and in Asian female patients [144,145]. An exception is represented by *NTRK* fusions that occur in NSCLC across sexes, ages, smoking histories and histologies [19].

The identification of driver alterations is clinically relevant since in oncogene-addicted subgroups immune checkpoint inhibitors (ICIs), even in presence of high levels of PD-L1 expression, are associated with poor outcomes [146,147,148,149,150]. Mechanisms underlying this phenomenon remain unclear, a possible explanation recognizes the lower tumor mutation burden (TMB) as responsible for ICIs’ modest response rates in this setting.

Globally, biomarker-driven therapy with innovative drugs produces response rates comparable to those seen with other targeted therapies, including osimertinib in EGFR-mutant NSCLC (80%), alectinib in ALK-positive NSCLC (83%), and entrectinib (77%) and crizotinib (72%) in ROS1 fusion-positive NSCLC [16,151,152]. These novel targeted agents are also characterized by intracranial activity, allowing disease control in patients with CNS metastases, a well-known poor prognostic factor. Finally, recent evidence suggests that *MET* amplification, *NTRK* rearrangements and *HER2* mutations might have a role in acquired resistance to EGFR-TKI [29,153]. Targeting these alterations could be an effective strategy to overcome resistance, increasing the number of therapeutic options after progression, and possibly delaying chemotherapy initiation.

## 4. Conclusions

NSCLC represents the paradigm of how biomarker-driven therapy can change disease natural history. Unfortunately, the percentage of patients carrying a potentially targetable genetic alteration is low. An extensive understanding of disease biology and broad tumor molecular profiling are essential steps in order to discover novel druggable targets, thus expanding the proportion of patients eligible for targeted agents. Future research should also focus on unraveling the mechanisms of acquired resistance, defining the optimal therapeutic sequence and exploring the potential efficacy of combination treatments.

## Figures and Tables

**Table 1:** Clinical activity of currently FDA approved targeted agents.

Clinical Trial	Trial Type	Driver Mutation	Treatment Arms	Clinical Outcomes	Most Frequent AEs (All Grades)
**GEOMETRY mono-1**	Phase 2, multicenter, multi-cohort, single-arm, non-randomized, open-label study	METex14 skipping mutation	Capmatinib	Pretreated pts: ORR 41%, DoR 9.7, mPFS 5.4 m. Treatment naïve: ORR 68%, DoR 12.6, mPFS 12.4 m.	Peripheral edema, nausea, vomiting, creatinine increase.
**VISION**	Phase 2, multicenter, multi-cohort, single-arm, non-randomized, open-label study	METex14 skipping mutation	Tepotinib	Combined biopsy: ORR by ICR 46%, DoR 11.1 m, mPFS 8.5 m. Liquid biopsy: ORR by ICR 48%, DoR 9.9 m, mPFS 8.5 m. Tissue biopsy: ORR by ICR 50%, DoR 15.7 m, mPFS 11 m.	Peripheral edema, nausea, diarrhea, creatinine increase.
**LIBRETTO-001**	Phase 1/2 trial, international, open-label study	RET fusion	Selpercatinib	ORR by ICR 64%, DoR 17.5 m, mPFS 18.4 m.	Diarrhea, AST increase, dry mouth, hypertension, fatigue.
**ARROW**	Phase 1/2, multi-cohort, international, open-label study	RET fusion	Pralsetinib	Pretreated: ORR 61%, DoR NR, mPFS 17.1 m. Treatment naïve: ORR 70%, DoR 9.0 m, 9.1 m.	AST/ALT increase, anemia, leucopenia, fatigue, constipation.
**LOXO-TRK-14001**	Phase 1/2, multi-cohort, international, open-label study	NTRK gene fusion	Larotrectinib *	Overall population: ORR 75%, DoR NR, mPFS NR. (7 lung cancer pts enrolled)	AST/ALT increase, anemia, neutropenia, weight increase.
**SCOUT/** **NAVIGATE**	Phase 1/2, multicenter, multi-cohort, single-arm, non-randomized, open-label study	NTRK gene fusion	Larotrectinib *	ORR 30%, DCR 70%, mPFS 18.3 m, mOS NR.	AST/ALT increase, leucopenia, neutropenia, vomiting.
**STARTRK-1, STARTRK-2, ALKA-372-001** **(Pooled analysis)**	Phase 1/2, multicenter, single-arm, non-randomized, open-label study	NTRK gene fusion, ROS1 rearrangement	Entrectinib	Overall population: ORR 59.3%, DoR 12.9, mPFS 12.9 m, mOS 23.9 m.	Fatigue, dysgeusia, paresthesia, nausea, myalgia.
**CodeBreak 100**	Phase 2, multicenter, international, single arm, open-label study	*KRAS^G12C^* mutation	Sotorasib	ORR by ICR 37.4%, DCR 80.5%, mPFS 6.7 m.	AST/ALT increase, leucopenia, anemia, diarrhea, myalgia, nausea, fatigue, hepatotoxicity, cough.

Abbreviations: ORR, objective response rate; pts, patients; mPFS, median progression-free survival; mOS, median overall survival; m, months; AEs, adverse events; DoR, duration of response; ICR, independent central review; AST, aspartate aminotransferase; ALT, alanine transferase; NR, not reached; DCR, disease control rate. * Larotrectinib received agnostic approval for NTRK fusion-positive tumors.

**Table 2:** A selection of clinical trials testing novel strategies to target MET altered NSCLC.

NCT Number	Gene Alteration	Experimental Drug	Phase	Study Design	Current Status
**NCT05015608**	MET amplification	Savolitinib	3	Randomized: savolitinib + osimertinib vs. pemetrexed + cisplatin/carboplatin	Recruiting
**NCT04338243**	MET amplification	Glumetinib	1/2	Single group assignment	Unknown
**NCT02435121**	MET amplification	SAR125844	2	Single group assignment	Completed
**NCT02544633**	MET mutation, amplification	MGCD265	2	Single group assignment	Completed
**NCT03175224**	METex14, amplification, fusion	APL-101	1/2	Single group assignment	Recruiting
**NCT04270591**	MET mutation, amplification	Glumetinib	1/2	Single group assignment	Active, not recruiting
**NCT02648724**	MET amplification	Sym015	1/2	Single group assignment	Completed
**NCT03539536**	MET amplification	Telisotuzumab vedotin	2	Single group assignment	Recruiting
**NCT05163249**	MET amplification	Savolitinib	2	Randomized: osimertinib ± savolitinib	Not yet recruiting
**NCT03993873**	MET mutation, amplification	TPX-0022	1/2	Single group assignment	Recruiting

**Table 3:** Trials testing novel agents targeting RET fusions in NSCLC.

NCT Number	Gene Alteration	Experimental Drug	Phase	Study Design	Current Status
**NCT04683250**	RET alterations	TAS0953/HM06	1/2	Sequential assignment	Recruiting
**NCT03784378**	RET mutation	RXDX-105	1	Single group assignment	Completed
**NCT03780517**	RET alterations	BOS172738	1	Sequential assignment	Active, not recruiting
**NCT04161391**	RET fusion, mutation	TPX-0046	1/2	Single group assignment	Recruiting
**NCT01877811**	RET rearrangement, fusion	RXDX-105	1	Single group assignment	Completed

**Table 4:** Trials testing novel agents or combination strategies for *KRAS* mutant NSCLC.

NCT Number	Gene Alteration	Experimental Drug	Phase	Study Design	Current Status
**NCT03170206**	*KRAS* mutation	Binimetinib plus palbociclib	1/2	Single group assignment	Recruiting
**NCT05374538**	*KRAS^G12C^* mutation	VIC-1911 plus sotorasib	1	Single group assignment	Not yet recruiting
**NCT04685135**	*KRAS^G12C^* mutation	Adagrasib	3	Randomized: adagrasib vs. docetaxel	Recruiting
**NCT04735068**	*KRAS* mutation	Binimetinib	2	Single group assignment	Recruiting
**NCT05132075**	*KRAS^G12C^* mutation	JDQ443	3	Randomized: JDQ443 vs. docetaxel	Recruiting
**NCT04967079**	*KRAS* mutation	Trametinib plus anlotinib	1	Single group assignment	Recruiting
**NCT04620330**	*KRAS* mutation	VS-6766	2	Randomized: VS-6766 ± defactinib	Recruiting
**NCT01933932**	*KRAS* mutation	Selumetinib	3	Randomized: docetaxel + selumetinib/placebo	Active, not recruiting
**NCT04613596**	*KRAS^G12C^* mutation	Adagrasib	2	Randomized: adagrasib ± pembrolizumab	Recruiting
**NCT03299088**	*KRAS* mutation	Trametinib plus pembrolizumab	1	Single group assignment	Active, not recruiting
**NCT03520842**	*KRAS* mutation	Regorafenib plus methotrexate	1	Single group assignment	Active, not recruiting
**NCT05276726**	*KRAS^G12C^* mutation	JAB 21822	1	Sequential assignment	Not yet recruiting
**NCT03681483**	*KRAS* mutation	RO5126766	1	Single group assignment	Active, not recruiting
**NCT02642042**	*KRAS* mutation	Trametinib plus docetaxel	2	Single group assignment	Active, not recruiting
**NCT03808558**	*KRAS* mutation	TVB-2640	2	Single group assignment	Recruiting
**NCT05375994**	*KRAS^G12C^* mutation	Defactinib plus adagrasib	1/2	Sequential assignment	Not yet recruiting
**NCT04449874**	*KRAS^G12C^* mutation	GDC-6036	2	Single group assignment	Recruiting
**NCT05379985**	*KRAS^G12^* mutation	RMC-6236	1	Single group assignment	Not yet recruiting
**NCT04965818**	*KRAS* mutation	Futibatinib and binimetinib	1/2	Single group assignment	Recruiting
**NCT04263090**	*KRAS* mutation	Rigosetib	1/2	Sequential assignment	Recruiting
**NCT04699188**	*KRAS^G12C^* mutation	JDQ443, TNO155, tislelizumab	1/2	Sequential assignment	Recruiting
**NCT05288205**	*KRAS^G12C^* mutation	JAB-21822, JAB-3312	1/2	Sequential assignment	Not yet recruiting
**NCT05358249**	*KRAS^G12C^* mutation	JDQ443	1/2	Sequential assignment	Not yet recruiting
**NCT05054725**	*KRAS^G12C^* mutation	RMC-4630 plus sotorasib	2	Sequential assignment	Recruiting
**NCT04956640**	*KRAS^G12C^* mutation	LY3537982	1	Single group assignment	Recruiting

## Data Availability

Not applicable.
